# Anti-inflammatory and immunomodulatory potential of lignans from *Artemisia cina*: Integrated quantum, docking, and cytokine expression studies

**DOI:** 10.1371/journal.pone.0349755

**Published:** 2026-05-28

**Authors:** Montserrath Abigail León Flores, Jocelyn Maza López, Sergio David Zavaleta Iglesias, Jorge Alfredo Cuéllar-Ordaz, Héctor Alejandro de la Cruz-Cruz, Maria Inés Nicolás Vázquez, Joel Martinez, Gerardo Ramírez Rico, Cynthia González-Ruiz, María Eugenia López-Arellano, Rosa Isabel Higuera-Piedrahita

**Affiliations:** 1 Laboratorio 3, Unidad de Investigación Multidisciplinaria, Facultad de Estudios Superiores Cuautitlán. Carr. Cuautitlán-Teoloyucan km 2.5, Cuautitlán Izcalli, Estado de México, México; 2 Centro Nacional de Investigación Disciplinaria en Salud Animal e Inocuidad, Instituto Nacional de Investigaciones Forestales, Agrícolas y Pecuarias. Carr. Fed. Cuernavaca-Cuautla, Jiutepec, Morelos, Mexico; 3 Departamento de Ciencias Químicas, Facultad de Estudios Superiores Cuautitlán Campo 1, Universidad Nacional Autónoma de México, Cuautitlán Izcalli, Estado de México, México; 4 Molecular Pathology Veterinary Laboratory, Unidad de Investigación Multidisciplinaria, Facultad de Estudios Superiores Cuautitlán. Carr. Cuautitlán-Teoloyucan km 2.5, Cuautitlán Izcalli, Estado de México, México; University of Buea, CAMEROON

## Abstract

Quantum, docking, and *in vitro* studies were performed to evaluate the immunomodulatory function of two lignans isolated from *Artemisia cina*—3-demethoxy-6-O-demethylisoguaiacin (**L1**) and norisoguaiacin (**L2**)—through individual molecular docking with cyclooxygenase-2 (COX-2) and their mixture for cytokine assays. The structures of **L1** and **L2** were optimized using density functional theory with the B3LYP/6–311++G(d,p) method. The optimized structures were then docked into the COX-2 active site, revealing slightly lower binding affinities (ΔG = −7.57 and −7.13 kcal/mol, respectively) compared with naproxen (−8.95 kcal/mol). The interactions of the ligands with Ser530 and Arg120 in the arachidonate-binding site predict a potential COX-2 inhibition through π-donor hydrogen interactions, along with several hydrophobic and π–π interactions. In the *in vitro* assays, both lignans significantly upregulated the anti-inflammatory cytokines interleukin (IL)-6, IL-4, and IL-13, which are involved in host protection against nematodes. By contrast, the chemokine CXCL8 was downregulated, indicating a reduction in inflammatory signaling. Moreover, the combination of **L1** and **L2** with lipopolysaccharide showed a synergistic effect, increasing the relative expression of IL-13 (212.8-fold), highlighting their immunomodulatory activity. These findings from in silico and in vitro assays suggest that the lignans could have potential anti-inflammatory effects, but direct evidence of COX-2 enzymatic inhibition is still lacking. Further in vivo studies are warranted to validate their therapeutic potential. Further *in vivo* studies are warranted to validate their therapeutic potential.

## Introduction

The infection process of *Haemonchus contortus* begins once the parasite enters the host and avoids detection by the host’s immune system. It accomplishes this through mimicry of the host’s immune antigens, which prevents activation of the innate immune system via Toll-like receptors (TLRs). As a result, the production of acute-phase proinflammatory substances—such as histamine and prostaglandins—is suppressed, along with cytokines (e.g., interleukin [IL]-4, IL-5, IL-10, and IL-13), mast cells, basophils, and eosinophils [1]. Moreover, activation of TLR-induced signaling is associated with an acute inflammatory response mediated by prostaglandins through the cyclooxygenase (COX) enzyme, as well as by high concentrations of histamine in the abomasal mucosa. Together, these factors contribute to parasite expulsion by promoting hypersecretion and hypermotility of the abomasum, which negatively affect the parasite’s fecundity and motility [2].

Once the parasite has established itself in the host, the presence of larvae induces infiltration of inflammatory cells and the replacement of hydrochloric acid–secreting cells with young, non-secretory cells. This leads to an increase in abomasal pH, which in turn reduces the conversion of pepsinogen to pepsin, decreases protein digestion, and increases mucosal permeability [1]. Therefore, regulating the inflammatory response mediated by COX could help alleviate the acute signs of gastrointestinal nematodes such as *H. contortus*.

Prostaglandin-endoperoxide synthase (EC 1.14.99.1), also known as COX, is responsible for synthesizing prostaglandins from arachidonic acid [3]. Two main isoforms have been identified: COX-1 and COX-2. COX-1 is constitutively expressed in all tissues and maintains basal prostaglandin levels. By contrast, COX-2 is an inducible enzyme whose expression is regulated by inflammatory cytokines, including tumor necrosis factor-alpha, nuclear factor kappa B, and IL-6. COX-2 activation is associated with the development and progression of several chronic inflammation–mediated diseases, including cancer, cardiovascular disease, and intestinal inflammation in humans [4]. Meanwhile, COX-1 maintains baseline prostaglandin levels and plays a key role in hydrochloric acid secretion, mucus production, mucosal blood flow, and the maintenance of mucosal integrity [5].

Increased arachidonic acid metabolism is closely related to sheep susceptibility to pathogen infections, such as the nematode *H. contortus*. For example, in the Suffolk breed [6], increased expression of proinflammatory genes related to eicosanoids as regulatory metabolites—such as *TLR4* and *COX2*—has been observed. Additionally, several genes involved in arachidonic acid metabolism are linked to resistance mechanisms against *H. contortus* in native sheep breeds from the Canary Islands, including arachidonate 5-lipoxygenase (*ALOX5*) and its activating protein (*ALOX5P*), prostaglandin reductase 1 (*PTGR1*), prostaglandin-endoperoxide synthase 1 (*COX1*), and thromboxane A synthase 1 (*TBXAS1*) [7].

Control of *H. contortus* in small ruminants has traditionally relied on chemical anthelmintics [8]. However, inappropriate and excessive use of these agents has contributed to the emergence of resistant parasites in several countries, including those where small ruminant farming is economically important, such as Mexico [8]. Consequently, our group has focused on exploring alternative control strategies. One option is the use of plants that produce bioactive compounds with anthelmintic activity. The *Artemisia* genus includes several species with both anthelmintic and anti-inflammatory properties, with *Artemisia cina* standing out [9–12].

*Artemisia cina* synthesizes various secondary metabolites, including sesquiterpenes, flavonoids [12], and lignans [13]. Our research group previously identified two lignans obtained from the n-hexane extract of *A. cina*: 3-demetoxy-6-O-demethylisoguaiacin and norisoguaiacin, both of which show strong antiparasitic activity [13].

Flores Jiménez et al. (2024) evaluated the acute toxicity of lignans obtained from *A. cina* using assays conducted according to OECD (2023) guidelines, demonstrating that these metabolites have a safety margin up to 200 times their therapeutic dose, permitting oral administration. Notably, these compounds induce overexpression of oxidative stress–related enzymes in the parasite, offering insight into their potential mechanism of action [14]. However, this mechanism must be further clarified through *in silico* studies such as docking (molecular coupling) and later confirmed through *in vitro* experiments.

Although our group previously established the antiparasitic activity of these lignans [13], their anti-inflammatory and immunomodulatory mechanisms have not been explored. Therefore, this study was performed to investigate, for the first time, the anti-inflammatory potential of lignans L1 and L2 by evaluating their binding to COX-2 through molecular docking and examining their effects on cytokine expression patterns in lymphocytes, which may partly explain their antiparasitic efficacy. It is important to note that while molecular docking can predict binding modes and affinities, it does not provide direct evidence of enzymatic inhibition; therefore, our docking results should be interpreted as hypothesis-generating.

## Materials and methods

### Plant material

Fresh aerial parts (leaves and stems) of *A. cina* were harvested during the pre-flowering stage. A total of 10 kg of plant material was purchased from Hunab® Laboratory. A voucher specimen was authenticated by Dr. Alejandro Torres-Montúfar and deposited in the herbarium of the Facultad de Estudios Superiores Cuautitlán (FES-C), Mexico. According to the supplier, the plants were cultivated under controlled conditions: 80% relative humidity, an average temperature of 24°C, and soil with a pH of 6.3.

### Obtaining of lignans

Ten kilograms of *A. cina* were macerated in 20 L of *n*-hexane for 48 h. The extract was concentrated in a rotary evaporator and lyophilized for use, and it was kept refrigerated prior to further processing. Lignans were obtained from the *n*-hexane extract after column chromatography using a gradient of *n*-hexane and ethyl acetate with decreasing concentrations (silica gel, from *n*-hexane–ethyl acetate 100:0 to *n*-hexane–EtOAc 0:100), with a total volume of 100 mL. Fraction 7 was separated and compared with lignan standards (3-demethoxy-6-O-demethylisoguaiacin and norisoguaiacin) by thin-layer chromatography (*n*-hexane:ethyl acetate at 7:3). The lignan structures were confirmed by correlation with previous work from our research group using nuclear magnetic resonance and electrospray ionization–mass spectrometry analyses [13]. The fraction was lyophilized and refrigerated for later use in cell culture. The isolation and identification of lignans followed the procedures reported by Higuera-Piedrahita [13].

### Quantum chemical calculations

The computational chemistry calculations were carried out using semi-empirical and density functional theory (DFT) methods to determine molecular stability. A set of conformers corresponding to local minima in potential energy was generated in the gas phase through random-search conformational analysis using the AM1 semi-empirical method, performed with the Spartan 06 program [15]. Minimum-energy conformers were then selected for Lignan 1 (**L1**) and Lignan 2 (**L2**), followed by geometry optimization at the DFT level [16,17]. The minimal energies and structures of the lignans were calculated using the density functional three-parameter hybrid model (DFT/B3LYP) [18,19] and the extended 6–311++G(d,p) basis set [20,21]. This basis set is a valence triple-ζ set augmented with d-polarization functions on heavy atoms (C and O), p-polarization functions on hydrogen atoms, and diffuse functions on all atoms. Geometry optimizations were initially performed with full relaxation of all internal degrees of freedom. The Gaussian 16 program [22,23] and GaussView 06 [22–24] were used to draw the input molecules and perform the DFT quantum calculations for the ground state of the compounds. A vibrational frequency calculation was also conducted to obtain theoretical infrared spectra in the gas phase using the same level of theory. All vibrational frequencies for each form of the molecules were positive, confirming the validity of the optimization.

### Docking studies

A docking study was carried out to predict the interaction between the lignan molecules and the COX-2 protein. Because docking simulations only model potential binding modes, the results are predictive and do not directly demonstrate functional inhibition. Docking studies provide a computational tool used to propose, at an atomic level, protein–ligand interactions [25]. AutoDock 4.2 software was used for the docking analyses; this algorithm keeps the macromolecule rigid while allowing flexibility in the ligand [26–29]. The program provides free-energy values and binding-pose correlations between docking simulations and corresponding experimental data [30]. The initial three-dimensional structures of **L1** and **L2** were generated computationally, and their geometries were fully optimized using DFT before docking, as described in the Quantum Chemical Calculations section.

The three-dimensional structure of COX-2 was retrieved from the Protein Data Bank (PDB ID: 3NT1). Water molecules and other ligands were removed. A grid-based procedure was used to prepare the structural inputs and define all binding sites. A rectangular lattice (50 × 50 × 50 Å), with points separated by 0.375 Å, was centered on the COX-2 catalytic site and arachidonate-binding site (Arg120, Tyr385, and Ser530) [31], which was kept rigid. The COX-2 enzyme was docked with a naproxen molecule to validate the docking procedure. All docking simulations were performed using the hybrid Lamarckian genetic algorithm with an initial population of 100 randomly placed individuals and a maximum of 1.0 × 10^7^ energy evaluations. All other parameters were kept at their default settings.

### Relative expression of cytokines using peripheral blood polymorphonuclear cell (PBMC) *in vitro* assays

#### PBMC assays.

Cell cultures were performed as described by Maza-Lopez et al. [32]. PBMCs were collected from four young cattle following NOM-062-ZOO-1999 for the care and use of experimental animals. The ethical protocol for animal use was added in supplementary material. Blood was obtained by jugular venipuncture; the bull was restrained in a holding pen, and the procedure was painless and lasted approximately 1 minute. The experimental protocol was supervised by the Internal Committee for Care and Use of Experimental Animals (CICUAE-FESC) of the Facultad de Estudios Superiores Cuautitlán, Universidad Nacional Autónoma de México, under protocol number C24_25. PBMCs separation was performed using density gradients with the commercial reagent Lymphoprep™ (Axis Shield, USA), following the manufacturer’s instructions. Briefly, PBMCs were washed with phosphate-buffered saline (pH 7.4), centrifuged, and resuspended in RPMI + HEPES culture medium (Gibco, USA) supplemented with 10% fetal bovine serum (BYproducts, Mexico) and 1% antibiotic–antimycotic solution (Gibco, USA). Dilutions were prepared for trypan blue staining to differentiate live and dead cells. A total of 500,000 viable cells per well were seeded into 96-well plates. PBMCs were then exposed to six treatments with three replicates for each one: negative control with RPMI+HEPES media (no treatment), positive control (lipopolysaccharide [LPS] at 1 µg/mL), lignans mixture at 1 µg/mL (3-demethoxy-6-O-demethylisoguaiacin (**L1**) at 60% and norisoguaiacin (**L2**) at 40%), lignans mixture at 10 µg/mL, lignans mixture with LPS at 10 µg/mL, and lignans mixture with LPS at 1 µg/mL for 24 h at 37°C in an atmosphere of 5% CO_2_.

Cell viability was assessed using the CellTiter 96® Aqueous One Solution Cell Proliferation Assay (Promega, USA), a MTS-based colorimetric method. Briefly, 20 μL of the MTS reagent was directly added in each well and incubated for two hours at room temperature for all experimental groups. Then, the MTS reaction with the PBMCs number was measured using a Microplate ELISA reader (Bio-Rad, iMark^TM^, USA) at 490 nm to analyze the corresponding data per group to perform the statistic analysis [32].

The following formula was used to quantify cell viability:


$$\begin{array}{c} \text{Cell viability }(\%)=\;\frac{\text{ Cells stimulated with lignans X 100}}{\text{Cells without treatment }(\text{Negative control})} \end{array}$$

### RNA extraction and reverse transcription

The molecular studies were performed using 10x10^6^ PBMCs per each experimental group in order to reach 2.0 of RNA purity. Total RNA was collected using the commercial reagent TRIzol™ (Invitrogen, USA), following the protocol described by Maza-Lopez et al. [32]. RNA concentration and purity were measured by spectrophotometry at 260 nm and 280 nm (NanoDrop-1000; Thermo Scientific, USA). To verify RNA integrity, a 3% agarose gel electrophoresis was run for 30 minutes at 60 V. The extracted total RNA was treated with the RQ1 RNase-Free DNase® kit (Promega, USA) to remove any traces of genomic DNA. Complementary DNA (cDNA) synthesis was performed using the ImProm-II Reverse Transcription System (Promega, USA) according to the manufacturer’s instructions. Reverse-transcription quantitative real-time polymerase chain reaction (RT-qPCR) assays were conducted on a Rotor-Gene Corbett 600 (Qiagen, Hilden, Germany) in a final volume of 20 µL, containing 10 µL of GoTaq® qPCR Master Mix (2X), 1 µL of primers (forward/reverse mix, 20 µM), 2 µL of cDNA (diluted 1:10), and 7 µL of nuclease-free water.

### RT-qPCR assay

Commercial synthetic oligonucleotides (Qiagen Sciences, MD, USA) were used to perform the RT-qPCR assays [33]. Cytokine genes included *IL-4*, *IL-6*, *IL-13*, chemokine *CXCL8*, and *β2-microglobulin*, along with the reference genes *actin* and *glyceraldehyde-3-phosphate dehydrogenase* (*GAPDH*). RT-qPCR reactions were run on a Rotor-Gene Corbett 600 (Qiagen, Hilden, Germany) using GoTaq® qPCR Master Mix (Promega, USA). A negative control without cDNA was included to rule out RNA contamination.

The amplification protocol consisted of an initial denaturation at 95°C for 2 minutes, followed by 40 cycles of denaturation at 95°C for 15 seconds and annealing/extension at 60°C for 1 minute. Fold-change values for each gene were determined using the comparative double delta C_T_ (2^ΔΔCT^) method. All data were analyzed relative to untreated cells (control group), using *β-tubulin* and *GAPDH* as reference genes. Relative expression analysis for each target gene was performed using the Qiagen® Gene Data Analysis platform (https://geneglobe.qiagen.com/us/analyze) (revised 12 June 2025) with Student’s *t*-test (p ≤ 0.05). The primers for the *IL* genes are listed in Table 1.

**Table 1:** Oligonucleotide sequences used for RT-qPCR analysis.

Gene Symbol	Gene Name	Accession Number	Primer Sequence (5’ → 3’)	Amplicon Size (bp)	Reference
***IL4***	Interleukin-4	NM_173921.2	F: CCTCACAGCAACGAAGAACA R: TCCAACGTAAGACGGTGCAT	85	[33]
***IL6***	Interleukin-6	NM_173923.2	F: CTGGCAGAAAACAACCTGAAC R: GCATCACCTTTGGCATCTT	74	[33]
***IL13***	Interleukin-13	NM_001077828.1	F: CAGCATGGTATGGAGCGTCA R: AGGCCATGTTGCAGAGGTTC	89	[34]
***CXCL8***	C-X-C motif chemokine ligand 8	NM_173925.2	F: CTGGCAGTTTTGCCAAGGAG R: TTTGGGGTGGAAAGGTTTGG	91	[34]
***B2M***	Beta-2-microglobulin	NM_173893.3	F: ACCGTGATCTTTCTGGTGCTT R: CAGTTCAGTATGTTCGGCTTC	65	[34]
***ACTB***	Beta-actin	NM_173979.3	F: CCACGAAACTACCTTCAACTCC R: GCATACAGGGACAGCACAGC	107	[33]
***GAPDH***	Glyceraldehyde-3-phosphate dehydrogenase	NM_001034034.2	F: GGGTCATCATCTCTGCACCT R: GGTCATAAGTCCCTCCACGA	176	[33]
***TUBB***	Beta-tubulin class I	NM_001077893.2	F: TGCCTTTGTGCACTTCACAC R: CCGGACACAATGGTATTGGA	72	[34]

F: forward primer, R: reverse primer. *ACTB, GAPDH,* and *TUBB* were used as reference genes for normalization. Primer sequences for *IL13, CXCL8, B2M,* and *TUBB* were obtained from the manufacturer’s (Qiagen) QuantiTect Primer Assay catalog [34].

### Statistical analysis

RT-qPCR data, expressed as fold-change (2^ΔΔCT^), were analyzed to determine statistical differences between treatment groups and the untreated control. An unpaired Student’s *t*-test was used for direct comparisons. For multiple comparisons among all treatment groups, one-way analysis of variance was performed, followed by Tukey’s post hoc test. Cell viability (MTS) data were also analyzed by one-way analysis of variance with Tukey’s test. Differences were considered statistically significant at p ≤ 0.05. All analyses were carried out using GraphPad Prism software (version 9.0.0).

## Results

### Structures and optimization of lignans

**Fig 1:**
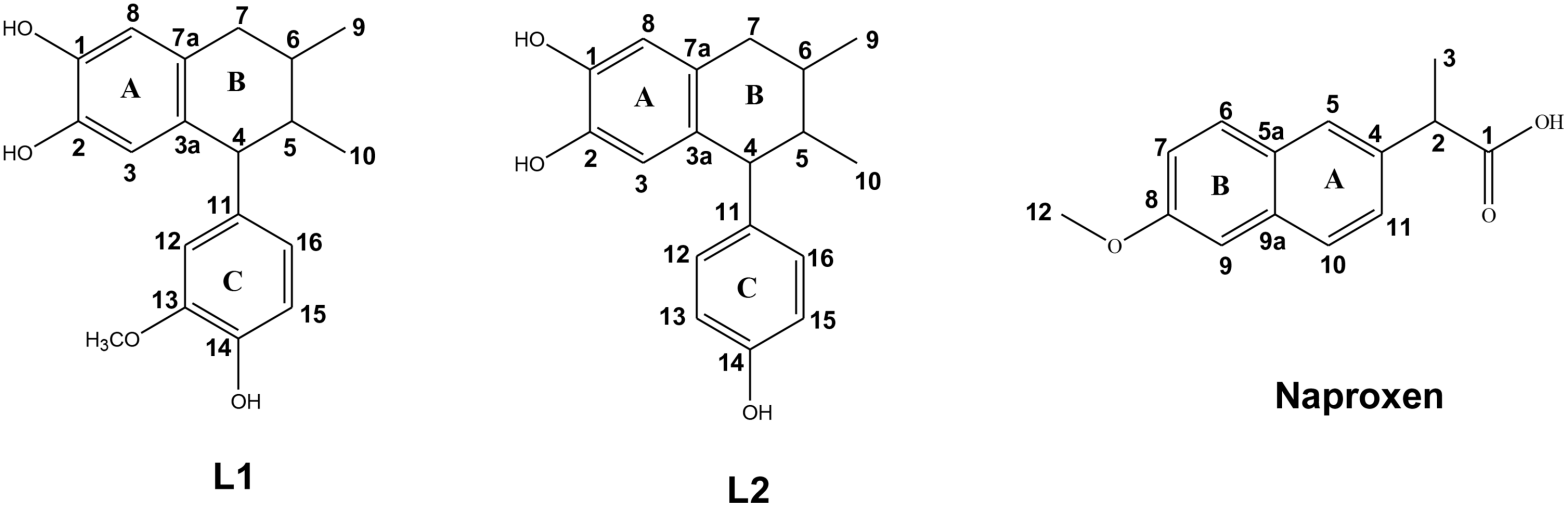
Structures and numeration of L1, L2, and naproxen.

To provide a clearer context for this study, Fig 1 shows the structures and assigned atom enumeration of the lignans (**L1** and **L2**) as well as naproxen.

**Fig 2:**
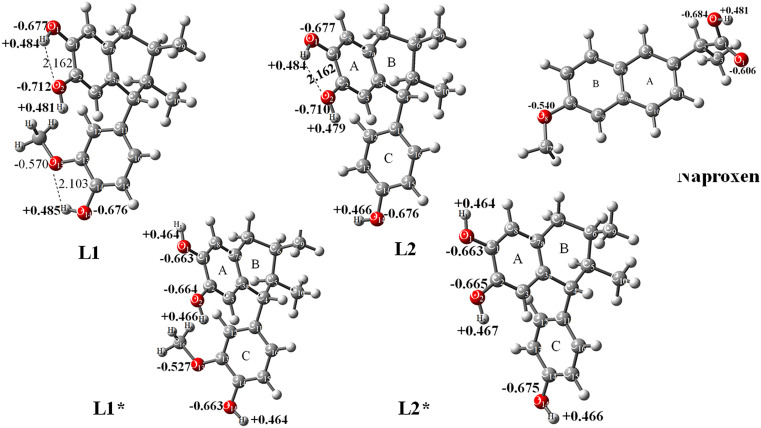
Optimized molecules: L1 and L2, with intramolecular hydrogen bond; L1* and L2* without intramolecular hydrogen bond, and naproxen. Bond lengths of intramolecular hydrogen bonding (Å) and natural populations analysis (charge, bold character, in e^-^) are indicated; at B3LYP/6-311++G(d,p) level of calculation.

The conformers labeled L1* and L2* represent alternative low-energy structures of **L1** and **L2**, respectively, identified during conformational analysis; these forms lack the specific intramolecular hydrogen bonds present in the primary conformers. At the quantum chemistry level, the conformers with and without intramolecular hydrogen bonding showed optimized energies of −1038.45731 Hartrees for **L1** and −1038.442932 Hartrees for L1*, with a difference of 9.0 kcal/mol. **L2** displayed an electronic energy of −923.898455 Hartrees, while L2* showed −923.891618 Hartrees, with a difference of 4.3 kcal/mol. These results indicate that the conformers exist in equilibrium (Fig 2).

### COX-2 binding affinity

#### Docking studies.

To determine both the binding mode and the free energy of binding (ΔG) of the studied compounds to human COX-2, docking studies were performed using AutoDock 4.2. Both conformers—those with intramolecular hydrogen bonds and those without—were evaluated at the docking level. The conformers with the most favorable ΔG values are reported here: **L1** and **L2** showed binding energies of −7.57 and −7.13 kcal/mol, respectively. For the non–intramolecular hydrogen-bonded conformers, L1* displayed a ΔG of −7.50 kcal/mol, and L2* showed −7.27 kcal/mol. In addition, naproxen was included to validate the docking procedure and exhibited a ΔG of −8.95 kcal/mol. Human COX-2 is a homodimer consisting of 581 amino acids and contains three high-mannose oligosaccharides, one of which facilitates proper protein folding [35]. The binding modes of **L1** and **L2** (with intramolecular hydrogen interactions) and L1* and L2* (without such interactions) with COX-2 residues are shown in and Table 2.

**Table 2:** Interaction of amino acid residues of COX-2 with **L1**, **L2**, L1*, L2*, and naproxen.

L1	L1*	L2	L2*	Naproxen
Ser353	Ser353	Leu352	Leu352	Ala527
Ala527	Ala527	Ser353	Ser353	Leu352
Leu352	Leu352	Ala527	Ala527	Val349
Val349	Val349	Leu531	Leu531	Gly526
Ser530	Ser530	Arg120	Arg120	Arg120
Trp387	Trp387	Tyr355	Tyr355	Fe518
Met522	Met522	Val523	Val523	Leu531
Leu384	Leu384			Leu384
Val523	Val523			Met522
				Trp387
				Tyr355
				Val523

Table 3 summarizes the free energy of binding (ΔG) for all docked ligands.

**Table 3:** Calculated free energy of binding (ΔG) for the interaction of lignans and naproxen with COX-2.

Compound	Binding Free Energy (ΔG, kcal/mol)
**L1**	−7.57
L1*	−7.50
**L2**	−7.13
L2*	−7.27
**Naproxen**	−8.95

**Note: L1** and **L2** are the primary conformers. L1* and L2* are the alternative conformers lacking the specific intramolecular hydrogen bonds identified in the conformational analysis.

**Fig 3:**
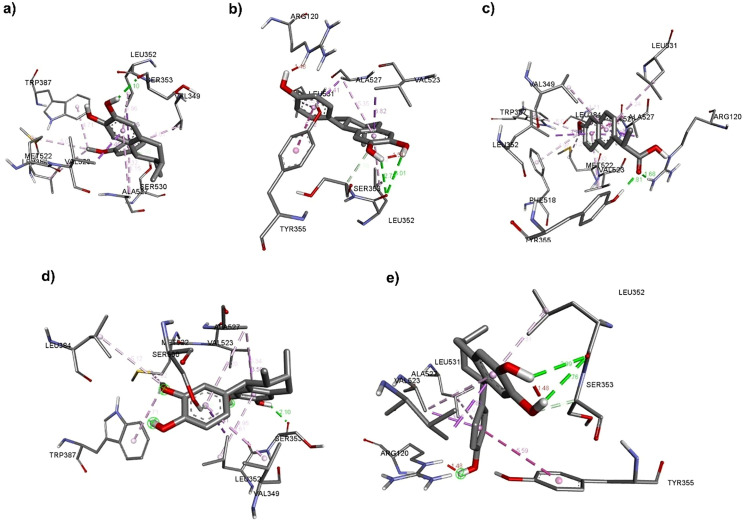
Amino acid interactions between L1, L2, naproxen, L1*, and L2*, obtained by docking studies.

Fig 3 (**L1**) shows the binding mode of the compounds studied within the COX-2 catalytic site. For **L1**, a hydrogen bond is formed between the hydroxyl group at position 2 and the amino acid residue Ser353, with a distance of 2.10 Å. Three π–sigma interactions are also observed: one between the A ring and Ala527, and two between the C ring and Val349 and Ala527. A π-donor hydrogen interaction appears between the C ring and Ser530. Finally, three alkyl–alkyl interactions are present between the methyl group of the methoxy substituent and the residues Met522, Trp387, and Leu384.

For **L2** (Fig 3), the residue Leu352 forms two hydrogen bonds with both hydroxyl groups on the A ring, at distances of 3.01 Å and 2.73 Å, respectively. A carbon–hydrogen bond is observed between the methine carbon of Ser353 and the oxygen of the hydroxyl group at position 2. In addition, two π–sigma interactions occur between the A ring and Val523, and between the C ring and Ala527; one π–alkyl interaction between the C ring and Leu531; and one π–π T-shaped interaction between the C ring and Tyr355. It is also important to note that Arg120 shows an unfavorable interaction with the hydroxyl group at position 14. This unfavorable interaction is likely due to the reduced availability of the lone pair on the secondary amino group of the guanidine moiety, which resonates with the imine moiety, thereby diminishing its ability to form a hydrogen bond with the hydroxyl hydrogen; nevertheless, the interaction persists.

For L1* (Fig 3), a hydrogen bond with Ser353 is observed at a distance of 2.10 Å. A π-donor hydrogen interaction forms between the C ring and Ser530. Two π–sigma interactions occur between the A ring and Val523, and between the C ring and Leu352, along with three additional π–sigma interactions involving the A and C rings with Ala527 and the C ring with Val349. Three hydrophobic interactions are noted between the methoxy group and the residues Trp387, Leu384, and Met522.

For L2* (Fig 3), two hydrogen bonds are formed with the amino acid residue Leu352. A carbon–hydrogen bond is also observed between the methine carbon of Ser353 and the oxygen of the hydroxyl group at position 2. Additionally, two π–sigma interactions occur: one between the A ring and Val523, and another between the C ring and Ala527. A π–π T-shaped interaction is present between the C ring and Tyr355. Three π–alkyl interactions are also observed: two involving the A ring with Leu352 and Ala527, and one involving the C ring with Leu531. As in **L2**, Arg120 displays an unfavorable interaction with the hydroxyl group at position 14. This unfavorable interaction is likely caused by the limited availability of the lone pair on the secondary amino group of the guanidine moiety, which resonates with the imine system, thereby reducing its ability to form a hydrogen bond with the hydroxyl hydrogen; nevertheless, the interaction persists.

For naproxen [36], which was docked into the active site of COX-2 (Fig 3), two hydrogen bonds are observed between the carboxylic acid moiety and the residues Arg120 and Tyr355, with distances of 1.68 Å and 1.81 Å, respectively. Naproxen also displays five π–alkyl interactions: between the A ring and Ala527, Val349, and Leu531, and between the B ring and Leu352, Ala527, Gly526, and Val523. One alkyl–alkyl interaction occurs between the methoxy substituent and Leu531.

Taken together, these observations show that **L1** and L1* share the same amino acid interaction pattern, and a similar trend occurs with **L2** and L2*. In both cases, hydrophobic interactions are concentrated in the C ring. For **L1** and L1*, these interactions are generated by the methoxy group, while in **L2** and L2*, the absence of this group results in fewer interactions. Moreover, considering that the conformers exist in equilibrium, the mixture of L1 and L2 could theoretically contribute to anti-inflammatory activity, as described below. However, these docking predictions need to be confirmed by enzymatic assays.

### Molecular electrostatic surface potential (MESP) of lignans

The MESP is useful for understanding the interactions and chemical properties of molecules. It serves as a valuable descriptor for predicting reactive sites for electrophilic and nucleophilic attacks, as well as for identifying potential hydrogen-bonding regions [37].

**Fig 4:**
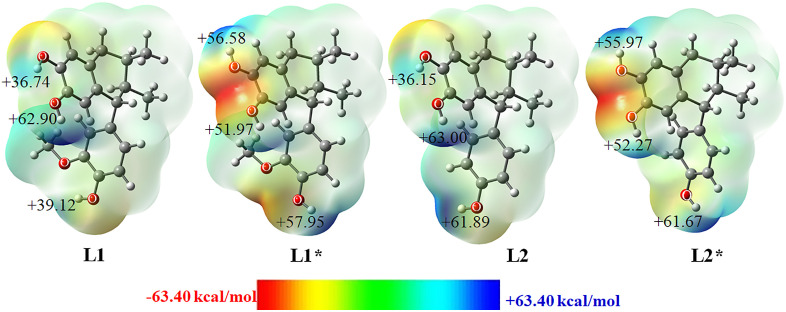
The structures optimized by DFT (B3LYP/6-311++G(d,p)) and 3D molecular electrostatic map of lignan on the 0.0004 a.u. contours of the electronic density of the molecules. The negative ESP regions are indicated in red, and the positive regions in blue.

The MESP map (Fig 4) shows a red-colored region surrounding the oxygen atoms (O1, O2, O13, and O14), indicating areas of negative potential associated with electrophilic attack. In addition, blue-colored regions appear around the hydrogen atoms of the hydroxyl groups, corresponding to positive potential and sites prone to nucleophilic attack. Green regions represent areas of neutral electrostatic potential. MESP analysis is frequently used to evaluate molecular electronic structure, reactivity, and structure–activity relationships [38]. In this context, the lignans establish hydrogen bonds through the hydrogen atoms of their hydroxyl groups with specific amino acid residues.

### Global reactivity descriptors

DFT defines concepts of chemical reactivity based on the electron density of the chemical system [38]. These descriptors include EHOMO, ELUMO, ΔE = ELUMO − EHOMO, ionization potential (IP), electron affinity (EA), electronegativity (χ), hardness (η) [39], and electrophilicity (ω) [40]. Together, they represent global reactivity parameters used to address qualitative aspects of chemical reactivity. All descriptors were calculated at the B3LYP/6–311++G(d,p) level using the following formulas:

- η = ½(I − A)
- χ = ½(I + A)
- ω = (µ^2^/2η)

Using the energies of the neutral, positively charged, and negatively charged species, vertical IP and vertical EA were determined as follows: IP = E_positive_ − E_neutral_, EA = E_neutral_ − E_negative_.

These indices measure the tendency of chemical species to gain or lose electrons. A good nucleophile is characterized by low ω values, whereas a good electrophile exhibits high ω values. EHOMO reflects the electron-donating ability, while ELUMO reflects the electron-withdrawing ability. The fact that all EHOMO and ELUMO energy values (and their neighboring orbitals) are negative indicates that all molecules are stable [41], as shown in Table 4.

**Table 4:** Global reactivity descriptors (eV) of the lignans calculated at the B3LYP/6-311++G(d,p) level in the gas phase.

Lignan	HOMO	LUMO	ΔE (E_LUMO_ − E_HOMO_)	IP	EA	η	χ	ω
L1	−5.77	−0.55	5.22	7.19	−0.22	3.71	3.49	1.64
L1*	−5.71	−0.52	5.19	7.13	−0.22	3.68	3.45	1.62
**L2**	−5.78	−0.69	5.09	7.31	−0.23	3.77	3.54	1.66
L2*	−5.71	−0.67	5.04	7.27	−0.22	3.74	3.52	1.66
Naproxen	−5.95	−1.35	4.60	7.58	−0.17	3.87	3.70	1.77

**Fig 5:**
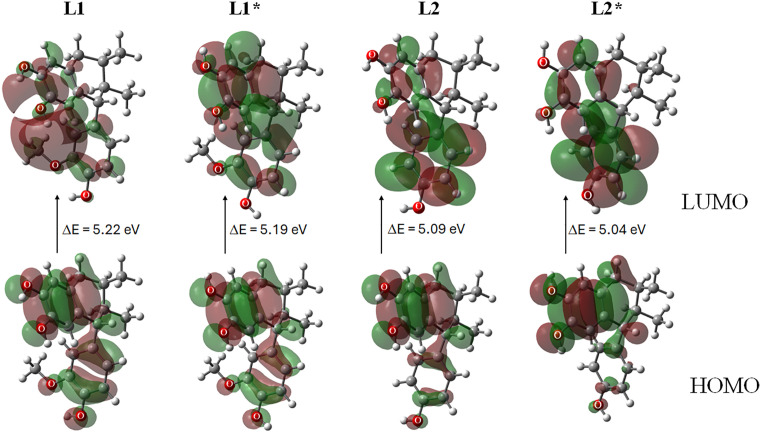
HOMO and LUMO contour plots for LI, LI*, L2 and L2* at the B3LYP/6-311++G(d.p) level.

The energy difference (ΔE = ELUMO − EHOMO) is a stability index that reflects the chemical reactivity of a molecule. Table 4 and Fig 5 show ΔE values ranging from 5.04 to 5.22 eV. **L2** and L2* (bearing a hydrogen atom at position 14) display ΔE values of 5.09 and 5.04 eV, making them slightly more reactive than **L1** and L1*, which contain an electron-donating substituent and show ΔE values of 5.22 and 5.19 eV.

Global hardness (η) is inversely related to reactivity: the harder the system, the lower its reactivity. Hardness reflects the ease of charge transfer within a molecule. Thus, **L2** and L2* are slightly harder than **L1** and L1*. Overall, the lignans exhibit similar hardness values, consistent with their HOMO–LUMO gaps and total energies.

The IP of a stable molecule is always positive, and both **L1** and **L2** show positive IP values. The stability trend suggested by the IP values is **L2** > L2* > **L1** > L1*. Conversely, all structures show negative EA values, indicating that energy is released upon forming the anion. **L1**, L1*, **L2**, and L2* exhibit similar EA values.

Electronegativity (χ) and electrophilicity (ω)—a measure of a system’s affinity for electron density—are shown in Table 4. According to these descriptors, the systems with the highest electron-density avidity are the **L2** and L2* lignans (Fig 5).

### Cell viability

The concentrations of the lignan mixture (1 and 10 µg/mL) were selected based on preliminary range-finding assays and prior studies involving similar bioactive lignans. These concentrations were subsequently confirmed to be non-cytotoxic to PBMCs in the MTS viability assay described below.

**Fig 6:**
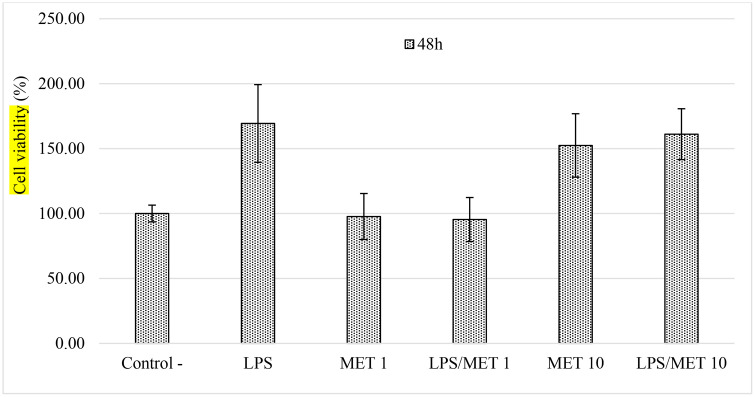
Cell viability of peripheral mononuclear cells exposed to the combination of two lignans (L1 and L2) with Lipopolysaccharide (LPS). The mixtures: LPS-lignans (LPS/MET1) at 1 and 10 µg/mL, lignans (MET) at 1 and 10 µg/mL, and a negative control.

Fig 6 shows lymphocyte viability following exposure to the lignan mixture at both concentrations. No significant reduction in cell proliferation was observed. Moreover, in the presence of LPS, the lignans maintained cellular proliferative capacity, indicating a non-cytotoxic profile for these compounds in PBMCs. In contrast, the lignans appeared to exert a protective effect against LPS-induced inflammation.

### mRNA expression patterns of cytokines in lymphocytes through cell culture

Table 5 shows the cytokine expression patterns from PBMCs exposed to the mixture of LPS and lignans. Significant cytokine regulation was observed for IL-4, IL-6, and IL-13, showing 4.45-fold, 6.40-fold, and 71.34-fold increases after 24 hours, respectively. By contrast, chemokine CXCL8 and β2-microglobulin were downregulated (0.19-fold and 0.02-fold, respectively). When lignans were combined with LPS, IL-13 displayed a 212.80-fold change, the highest expression level observed. These findings indicate that *A. cina* lignans modulate the inflammatory response by promoting cytokines, suggesting a possible mechanism for protective immune responses against the ruminant nematode *H. contortus*.

**Table 5:** Fold-change values for mRNA expression patterns of cytokines in polymorphonuclear cells exposed to LPS and lignans.

Genes	Fold change (compared with control group)					
	LPS		Lignans		Lignans + LPS	
	Fold change	p value	Fold change	p value	Fold change	p value
**IL-4**	**0.01**	**0.02**	**4.45**	0.20	**6.69**	0.06
**IL-6**	0.91	0.91	**6.40**	**0.05**	*0.43*	0.24
**CXCL8**	1.74	0.06	*0.19*	0.92	*0.31*	0.10
**IL-13**	**0.13**	0.07	**71.34**	0.17	**212.80**	**0.01**

*Italic* = downregulation; bold numbers= upregulation; bold text = p < 0.05.

**Fig 7:**
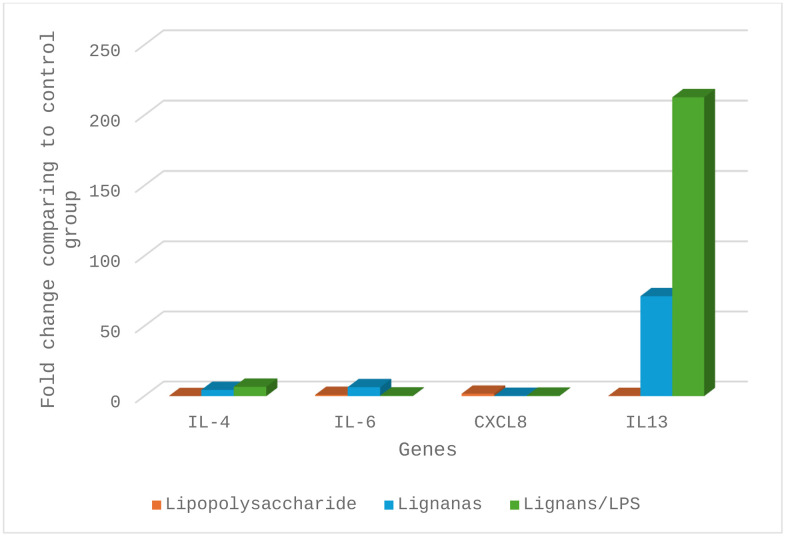
Fold change values for mRNA expression patterns of cytokines in polymorphonuclear cells *in vitro* assays exposed to lipopolysaccharide (LPS) and lignans.

Fig 7 shows the upregulation and downregulation patterns of these genes.

## Discussion

In recent papers from our group [42,43] employing computational studies, the affinity of COX-2 for natural product derivatives was proposed. Following stimulation by pro-inflammatory cytokines, COX-2 expression increases, resulting in elevated prostaglandin synthesis [44]. Consequently, COX-2 has been strongly linked to pathological inflammatory processes. The COX-2 active site consists of a hydrophobic channel through which nonsteroidal anti-inflammatory drugs (NSAIDs) are recognized [45].

Lignans **L1** and **L2** exhibit structural differences that influence their predicted interaction with COX-2. Molecular docking results showed that both lignans display similar predicted binding affinities. These findings suggest the potential of both lignans to act as COX-2 inhibitors in silico; however, naproxen shows the highest predicted binding affinity. Direct evidence of COX-2 inhibition would require enzymatic activity assays (e.g., using a purified COX-2 enzyme), yielding free energies of −7.57 and −7.13 kcal/mol, respectively. Although these values are lower in magnitude than those of naproxen (−8.95 kcal/mol), they still suggest meaningful interactions with the enzyme’s active site. **L1** has two intramolecular hydrogen bonds and good energetic stability; it interacts with Ser353 via hydrogen bonding. By contrast, **L2**, which contains only one intramolecular hydrogen bond, forms two hydrogen bonds with Leu352 (bifurcated acceptor).

The conformers without intramolecular hydrogen bonds showed similar binding energies (L1*, −7.50; L2*, −7.27 kcal/mol), indicating that although intramolecular hydrogen bonding influences ligand conformation, it does not drastically impact binding affinity. Both conformers of **L1** and **L2** interact via the hydroxyl hydrogen atoms with Ser353. These findings support the potential of both lignans as COX-2 inhibitors; however, naproxen remains the compound with the highest binding affinity.

Natural population analysis showed high negative charges on the hydroxyl oxygen atoms, indicating that these groups act as hydrogen-bond donors toward amino acid residues. The electrostatic potential map is consistent with this chemical behavior in both **L1** and **L2**.

Naproxen exhibits higher ionization potential, global hardness, electronegativity, and electrophilicity, as well as lower electron affinity and band gap values. Thus, it is a more stable molecule. Lignans **L1** and **L2** are comparatively less stable, yet they may still show pharmacological activity.

*In vitro* assays revealed that the lignans modulate cytokines with inflammatory activity that play important roles in T-cell differentiation and growth, including cytokines involved in repairing tissue damage caused by gastrointestinal nematodes, such as IL-4 and IL-13, which promote regulatory immune responses [46]. IL-4 contributes to helminth rejection by promoting immunoglobulin production (e.g., IgE). Both IL-4 and IL-13 have been associated with the inhibition of pro-inflammatory chemokines and cytokines, including tumor necrosis factor and IL-1β [47,48]. In the present study, no association was found between IL-4/IL-13 and CXCL8; however, the data suggest immune regulation of pro-inflammatory cells. Moreover, *A. cina* lignans did not induce strong inflammatory responses at low concentrations, and CXCL8 expression remained normalized or downregulated. These findings support the therapeutic potential of these lignans as modulators of inflammation and antiparasitic responses, particularly in chronic diseases caused by gastrointestinal nematodes such as *H. contortus*.

The molecular interaction of the lignans with COX-2, as evidenced by docking studies, suggests that these compounds may inhibit prostaglandin production, a mechanism similar to that reported for other flavonoids and lignans. For example, studies on *Vitex negundo* have shown that compounds such as vitexin and negundoside exert anti-inflammatory effects by regulating COX-2 and reducing pro-inflammatory cytokine production [49,50]. In our study, the strong overexpression of IL-13 (212.8-fold) in the presence of LPS and lignans suggests a potential synergistic effect, similar to observations in *V. negundo*, where flavonoids modulate immune responses through anti-inflammatory signaling pathways [51]. These parallels underscore the potential of plant secondary metabolites as multifunctional therapeutic agents.

The increase in IL-6 expression observed with the lignan treatment (Table 5) warrants careful analysis. IL-6 is a pleiotropic cytokine that can exert both pro-inflammatory and anti-inflammatory effects depending on the context; it is classically associated with acute-phase inflammation but also contributes to the resolution of inflammation and Th2 differentiation. Our observation of increased IL-6 together with upregulation of IL-4/IL-13 and downregulation of CXCL8 is consistent with a Th2-biased immunomodulatory profile rather than a purely anti-inflammatory state. However, this interpretation remains hypothetical without functional assays (e.g., blocking antibodies or cytokine neutralisation). This nuanced interpretation is consistent with the pleiotropic nature of IL-6 and the overall cytokine profile observed.

Beyond their anti-inflammatory activity, *A. cina* lignans displayed a promising safety profile, with no adverse effects on lymphocyte viability, consistent with findings in *V. negundo*, where lignan-rich extracts showed low cytotoxicity [52]. The ability of these compounds to induce apoptosis in parasites, as previously reported in *H. contortus*, suggests a dual mechanism—anti-inflammatory and antiparasitic. This effect resembles that of neolignans from *V. negundo*, which induce apoptosis in cancer cells through caspase activation and regulation of the Bcl-2/Bax pathway [50]. Future *in vivo* studies should validate these results, exploring optimal dosages and potential synergies with conventional treatments, similar to approaches used in inflammation models with standardized plant extracts [53].

The review by [54] highlights the chemical diversity of the Gesneriaceae family, including flavonoids, terpenoids, phenolic glucosides, and quinones. Many of these compounds correlate with traditional medicinal uses, such as treatment of fever, inflammation, and infections. For instance, anti-inflammatory flavonoids like naringenin [55] and aeschynanthoside D (182) in *Aeschynanthus bracteatus* support its traditional application in rheumatoid arthritis and postpartum care. Likewise, the antimicrobial properties of *Didymocarpus pedicellata* and *Kohleria deppeana* align with historical use against bacterial and fungal infections [56]. This synergy between phytochemistry and ethnobotany underscores the potential of Gesneriaceae as a source of bioactive compounds and highlights the need for further exploration of understudied species [56].

The findings from the *A. cina* lignans study align with the broader context of plant-derived bioactive compounds, as noted in the *Tanacetum* review [57]. Both studies emphasize the therapeutic potential of secondary metabolites—lignans in *A. cina* and sesquiterpenes and flavonoids in *Tanacetum*—in modulating inflammatory pathways [57]. While *Tanacetum* species demonstrate anti-inflammatory activity through COX-2 inhibition and cytokine regulation (e.g., IL-13 overexpression) [57], *A. cina* lignans similarly target COX-2 with binding affinities comparable to NSAIDs like naproxen, in addition to upregulating anti-inflammatory cytokines (IL-4, IL-13). Notably, both groups of metabolites show dual functionality: *Tanacetum* extracts are promising for migraine and oxidative stress, while *A. cina* lignans exhibit antiparasitic and anti-inflammatory actions. However, *Tanacetum* research includes more extensive *in vivo* validation, suggesting the need for similar translational studies on *A. cina* lignans [58]. Together, these findings highlight the versatility of plant metabolites in addressing inflammation and parasitic infections, advocating for integrated *in silico*, *in vitro*, and *in vivo* approaches to fully harness their therapeutic potential.

While our docking studies predict a potential interaction with COX-2, an important limitation of this work is the absence of in vitro enzymatic assays (e.g., COX-2 activity assays) to experimentally validate inhibition. Therefore, the term “COX-2 inhibitor” should only be applied to these lignans after such confirmatory studies. Future studies should include these assays to test the direct inhibitory effect.

## Conclusion

This study demonstrates that lignans exhibit predictive anti-inflammatory potential through predicted molecular interactions with COX-2, supported by in silico docking and in vitro cytokine modulation. The predicted binding energies of L1/L2 are weaker than those of naproxen; however, they suggest possible COX-2 binding. Based on these preliminary results, it is hypothesized that the molecules under study might exhibit anti-inflammatory activity in vivo, but this requires experimental validation. Moreover, these lignans upregulated anti-inflammatory cytokines (IL-4, IL-13) while downregulating pro-inflammatory markers (CXCL8), consistent with an immunomodulatory effect. Importantly, the compounds maintained lymphocyte viability, indicating low cytotoxicity. These findings position *A. cina* lignans as potential candidates for anti-inflammatory therapies, particularly in parasitic infections such as haemonchosis. Nevertheless, future vivo studies are essential to validate their therapeutic efficacy and safety.

## Supporting information

S1 File Supporting information.
(DOCX)
