# Supplementary material for: Anti-inflammatory and immunomodulatory potential of lignans from Artemisia cina: Integrated quantum, docking, and cytokine expression studies
Source: PLoS One. 2026 May 28;21(5):e0349755. doi: 10.1371/journal.pone.0349755 (PMC13218524; doi:10.1371/journal.pone.0349755)
Supplement: S1 File — Supporting information. (DOCX) [file pone.0349755.s001.docx]

Average Ct

| **Position** | **Symbol** | **AVG Ct** | | | | **Standard Deviation** | | | |
| --- | --- | --- | --- | --- | --- | --- | --- | --- | --- |
|  |  | **Control Group** | **Group 1** | **Group 2** | **Group 3** | **Control Group** | **Group 1** | **Group 2** | **Group 3** |
| A01 | IL4 | 20.53 | 29.37 | 20.27 | 18.73 | 4.985344 | 2.761099 | 1.379343 | 2.548083 |
| A02 | IL6 | 18.70 | 20.54 | 17.92 | 20.87 | 2.591134 | 1.786221 | 0.286618 | 3.877884 |
| A03 | CXCL8 | 14.65 | 15.56 | 18.92 | 17.31 | 1.577807 | 1.727415 | 1.416381 | 1.837755 |
| A04 | IL13 | 24.33 | 28.99 | 20.07 | 17.55 | 4.043577 | 2.748091 | 2.605910 | 1.756757 |
| A05 | B-micro | 10.43 | 11.13 | 18.18 | 16.15 | 0.635907 | 0.770142 | 0.534504 | 0.742890 |
| A06 | GAPDH | 13.44 | 15.26 | 18.64 | 18.23 | 0.968840 | 0.977907 | 1.371019 | 0.886327 |
| A07 | ACTINA | 19.95 | 21.66 | 21.85 | 20.90 | 2.833902 | 2.029403 | 1.697909 | 1.821511 |

Average Delta Ct

| **Position** | **Symbol** | **AVG Delta(Ct) (Ct(GOI) - Ave Ct(HKG))** | | | | **Standard Deviation** | | | |
| --- | --- | --- | --- | --- | --- | --- | --- | --- | --- |
|  |  | **Control Group** | **Group 1** | **Group 2** | **Group 3** | **Control Group** | **Group 1** | **Group 2** | **Group 3** |
| A01 | IL4 | 0.57 | 7.70 | -1.58 | -2.17 | 3.018919 | 4.610214 | 2.446924 | 4.281734 |
| A02 | IL6 | -1.26 | -1.12 | -3.94 | -0.04 | 2.607234 | 3.711748 | 1.698404 | 4.854053 |
| A03 | CXCL8 | -5.31 | -6.11 | -2.93 | -3.60 | 1.743909 | 2.879441 | 2.512441 | 3.419967 |
| A04 | IL13 | 4.38 | 7.33 | -1.78 | -3.36 | 2.611164 | 4.667221 | 3.490817 | 3.077040 |
| A05 | B-micro | -9.53 | -10.53 | -3.67 | -4.75 | 2.904391 | 2.283406 | 1.712207 | 2.214492 |
| A06 | GAPDH | -6.51 | -6.40 | -3.21 | -2.67 | 3.071165 | 2.393872 | 2.030444 | 2.033841 |
| A07 | ACTINA | 0.00 | 0.00 | 0.00 | 0.00 | 0.000000 | 0.000000 | 0.000000 | 0.000000 |

**2^(-Avg.(Delta(Ct))**

| **Position** | **Symbol** | **2^(-Avg.(Delta(Ct))** | | | |
| --- | --- | --- | --- | --- | --- |
|  |  | **Control Group** | **Group 1** | **Group 2** | **Group 3** |
| A01 | IL4 | 0.672580 | 0.004798 | 2.992002 | 4.500234 |
| A02 | IL6 | 2.391271 | 2.178497 | 15.301016 | 1.024951 |
| A03 | CXCL8 | 39.609588 | 68.858152 | 7.626976 | 12.097748 |
| A04 | IL13 | 0.048138 | 0.006215 | 3.434262 | 10.243712 |
| A05 | B-micro | 738.722592 | 1480.862753 | 12.767857 | 26.970929 |
| A06 | GAPDH | 91.420414 | 84.448506 | 9.282056 | 6.369195 |
| A07 | ACTINA | 1.000000 | 1.000000 | 1.000000 | 1.000000 |
